# Endoscopic Submucosal Dissection of Gastric Epithelial Neoplasms after Partial Gastrectomy: A Single-Center Experience

**DOI:** 10.1155/2017/6395283

**Published:** 2017-05-16

**Authors:** Byeong Gu Song, Gwang Ha Kim, Bong Eun Lee, Hye Kyung Jeon, Dong Hoon Baek, Geun Am Song

**Affiliations:** Department of Internal Medicine, Pusan National University School of Medicine and Biomedical Research Institute, Pusan National University Hospital, Busan 49241, Republic of Korea

## Abstract

**Aims:**

To investigate the feasibility and safety of endoscopic submucosal dissection (ESD) of gastric epithelial neoplasms in the remnant stomach (GEN-RS) after various types of partial gastrectomy.

**Methods:**

This study included 29 patients (31 lesions) who underwent ESD for GEN-RS between March 2006 and August 2016. Clinicopathologic data were retrieved retrospectively to assess the therapeutic ESD outcomes, including en bloc and complete resection rates and procedure-related adverse events.

**Results:**

The en bloc, complete, and curative resection rates were 90%, 77%, and 71%, respectively. The types of previous gastrectomy, tumor size, macroscopic type, and tumor histology were not associated with incomplete resection. Only tumors involving the suture lines from the prior partial gastrectomy were significantly associated with incomplete resection. The procedure-related bleeding and perforation rates were 6% and 3%, respectively; none of the adverse events required surgical intervention. During a median follow-up period of 25 months (range, 6–58 months), there was no recurrence in any case.

**Conclusions:**

ESD is a safe and feasible treatment for GEN-RS regardless of the previous gastrectomy type. However, the complete resection rate decreases for lesions involving the suture lines.

## 1. Introduction

The incidence of gastric cancer in the remnant stomach reportedly comprises 1-2% of all gastric cancers [1]; however, this incidence has been increasing in conjunction with increasing survival rates following gastrectomy due to gastric cancer. Furthermore, advances in diagnostic technology and periodic postgastrectomy surveillance have enabled the early detection of gastric epithelial neoplasms (GENs), such as early gastric cancer (EGC) and adenoma. In fact, metachronous gastric cancers occur in 0.6–3.0% of patients who undergo partial gastrectomy [2–4]. However, there is little information regarding the optimal treatment of GEN in the remnant stomach (GEN-RS).

Endoscopic submucosal dissection (ESD) is a widely accepted treatment modality for premalignant lesions and early cancers in the stomach [5, 6]. Similarly, ESD has been considered an effective treatment modality for GEN-RS because ESD can preserve the remnant stomach, leading to better patient quality of life [7]. ESD for GEN-RS, nevertheless, is notorious for its procedural difficulty because of the narrow inner space and the severe fibrosis along the suture lines [8]. In addition, massive postoperative adhesions around the remnant stomach make ESD of the tumors in the remnant stomach more difficult than that of the tumors in the whole stomach [8].

Several recent studies showed that ESD is an effective and safe treatment modality for GEN-RS after distal gastrectomy [9–13]. However, few studies have reported the therapeutic outcomes of ESD for GEN-RS after other types of partial gastrectomy [10, 14]. Therefore, we investigated the feasibility and safety of ESD for GEN-RS after various types of partial gastrectomy and also investigated the factors predicting incomplete resection.

## 2. Methods

### 2.1. Patients

We retrospectively analyzed our database of all patients who underwent gastric ESD at the Pusan National University Hospital (Busan, Korea) between March 2006 and August 2016. Thirty-one GEN-RS lesions were resected, using ESD, from 29 patients who had undergone various types of partial gastrectomy. The inclusion criteria were as follows: GEN-RS after partial gastrectomy, regardless of the type of the surgery; endoscopic morphology characteristics of a superficial neoplastic lesion, as described by the *Japanese Classification of Gastric Carcinoma* [15]; and a preprocedural biopsy indicating adenoma or adenocarcinoma. All cancer patients underwent abdominal computed tomography (CT), before ESD, to determine the presence of lymph node or distant metastases. Additionally, endoscopic ultrasonography was performed to rule out submucosal invasion in most cancer cases. All patients agreed to undergo ESD after receiving an explanation of the risks and benefits, including ESD-associated adverse events and the possible necessity of additional surgical treatment. Written informed consent was obtained from all patients before ESD, and the study protocol was reviewed and approved by the Institutional Review Board of the Pusan National University Hospital (E-1611-002-048).

### 2.2. Endoscopic Submucosal Dissection

**Figure 1:**
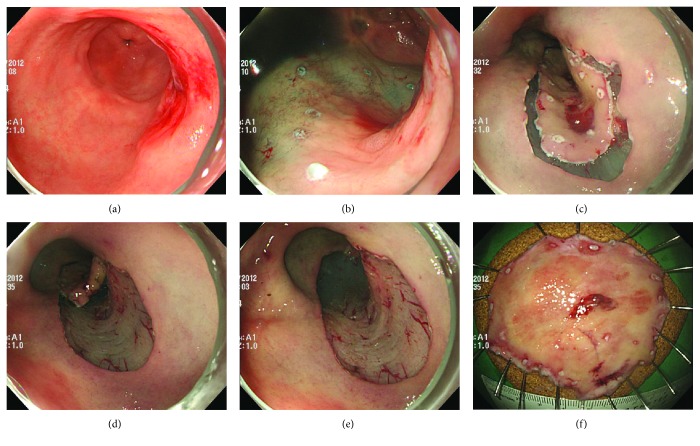
Endoscopic submucosal dissection for early gastric cancer in the gastric conduit, after esophagectomy. (a) A slightly depressed lesion is observed in the gastric conduit. (b) Circumferential marking is performed around the tumor using argon plasma coagulation. (c) A circumferential mucosal incision is made outside the marking dots with an electrosurgical knife. (d) Submucosal dissection is performed with an electrosurgical knife. (e) The lesion is completely removed. (f) Resected specimen.

ESD procedures were carried out by three experienced endoscopists using a single-channel endoscope (GIF-H260 or GIF-Q260; Olympus, Tokyo, Japan) [16]. All patients underwent ESD under conscious sedation, with cardiorespiratory monitoring. For sedation, 5–10 mg of midazolam and 25 mg of meperidine were administered intravenously; intraprocedural propofol was administered, as required. Argon plasma coagulation was used to mark the borders of the lesion, which had been identified using conventional endoscopy or chromoendoscopy with the application of an indigo carmine solution. After marking, a saline solution (0.9% saline with a small amount of epinephrine and indigo carmine) was injected into the submucosal layer around the lesion to lift the lesion off the muscular layer. A circumferential mucosal incision was made outside the marking dots with an IT knife (Olympus) and/or Flex knife (Olympus). Then, submucosal dissection was performed, with these knives, to completely remove the lesion (Figure 1). A high-frequency electrosurgical current generator (Erbotom VIO 300D; ERBE, Tübingen, Germany) was used during marking, mucosal incision, submucosal dissection, and hemostasis.

On the following day, all patients underwent postprocedural chest radiography and second-look endoscopy to detect any perforation or bleeding. Proton pump inhibitors and sucralfate were administered to relieve pain, prevent procedure-related bleeding, and promote ulcer healing. Patients without serious symptoms or adverse events were permitted to start food intake the day after the procedure and were discharged within 3-4 days.

### 2.3. Histopathologic Evaluation

The macroscopic findings of the lesions were categorized as protruding (I), nonprotruding and nonexcavated (II), or excavated (III). Type II lesions were subclassified as slightly elevated (IIa), flat (IIb), or slightly depressed (IIc). All lesions were also classified as elevated (I, IIa) or flat/depressed (IIb, IIc, III) types. Resected specimens were fixed in formalin and serially sectioned at 2 mm intervals to assess tumor involvement in the horizontal and vertical margins. The tumor size, depth of invasion, degree of differentiation, and lymphovascular invasion were evaluated microscopically by an expert gastrointestinal pathologist, according to the *Japanese Classification of Gastric Carcinoma* [15].

### 2.4. Outcome Parameters

The primary outcome was successful resection, including the rates of en bloc, complete, and curative resection. The secondary outcomes were procedure time, procedure-related adverse events, and local recurrence rate. En bloc resection was defined as tumor resection in a single piece, whereas complete resection was defined as successful en bloc resection with both horizontal and vertical margins histologically free of tumors. Curative resection was defined as a complete resection that fulfilled the following criteria [15]: (1) mucosal cancer, differentiated-type adenocarcinoma, no lymphovascular invasion, without ulceration, irrespective of tumor size; (2) mucosal cancer, differentiated-type adenocarcinoma, no lymphovascular invasion, with ulceration, tumor size ≤3 cm; (3) minute submucosal cancer invasion ≤500 *μ*m, differentiated-type adenocarcinoma, no lymphovascular invasion, tumor size ≤3 cm; or (4) mucosal cancer, undifferentiated-type adenocarcinoma, no lymphovascular invasion, without ulceration, tumor size ≤2 cm.

Procedure time was defined as the time from the initiation of the marking to the complete removal of the tumor. Procedure-related bleeding was defined as endoscopically proven bleeding within 24 hours, clinical evidence of melena or hematemesis, or massive bleeding requiring transfusion [17]. Successful endoscopic hemostasis of intraprocedural bleeding was not regarded as procedure-related bleeding. Perforations were endoscopically diagnosed during the procedure or by the presence of free air in the post-ESD plain chest radiography.

### 2.5. Follow-Up

In cases of curative resection, follow-up endoscopy was conducted 6 months after ESD and annually, thereafter. In cancer cases with curative resection, abdominal CT, chest radiography, and laboratory measurements of the tumor markers were performed 6 months after ESD and annually, thereafter. In cancer cases with noncurative resection, such as those with a positive vertical margin or deep submucosal invasion, an additional gastrectomy and lymph node dissection was recommended. However, for patients who refused a surgical operation, follow-up endoscopy with biopsies and abdominal CT were conducted 1-2 months and 4–6 months after ESD, respectively.

### 2.6. Statistical Analysis

Variables are expressed as medians or ranges and simple proportions. Statistical significance was evaluated using the *χ*^2^ test or Fisher's exact test for categorical variables. A *P* value of <0.05 was considered statistically significant. The statistical calculations were performed using SPSS version 21.0 for Windows software (SPSS Inc., Chicago, IL, USA).

## 3. Results

### 3.1. Baseline Characteristics of Patients with Gastric Epithelial Neoplasia in the Remnant Stomach

The clinicopathologic characteristics of the 29 patients (31 GEN-RS) are summarized in Table 1. The patients included 24 males and 5 females with a median age of 69 years. The types of previous gastrectomy included distal gastrectomy (*n* = 20) and gastric conduit (*n* = 9). The patients underwent surgery for cancer (17 patients for gastric cancer and 8 patients for esophageal cancer) or for benign diseases (3 patients for peptic ulcer bleeding and 1 patient for corrosive esophagitis). The median tumor size was 15 mm (range, 5–40 mm); the tumor sizes were ≤20 mm in 15 lesions and >20 mm in 14 lesions. Macroscopically, 19 lesions were elevated, 3 were flat, and 9 were depressed. Four lesions involved the suture lines, including the anastomosis site. The pathologic diagnoses of the lesions included 9 adenomas and 22 cancers (differentiated-to-undifferentiated-type adenocarcinoma, 16 : 6). Of the 22 cancers, 12 were mucosal cancers and 10 were submucosal cancers.

### 3.2. Outcomes of Endoscopic Submucosal Dissection

Table 2 shows the therapeutic outcomes of ESD for GEN-RS. The en bloc resection rate was 90% (28/31). Piecemeal resection occurred for 3 early cancers; the pathologic results indicated positive margin involvement and deep submucosal invasion. Of the 28 en bloc resected lesions, 4 had positive margins (horizontal involvement with the tumor cells in 2 and vertical involvement with tumor cells in 2). Therefore, the complete resection rate was 77% (24/31). Of the 24 completely resected tumors, deep submucosal invasion (>500 *μ*m from the muscularis mucosa) was found in 2 early cancer cases. As a result, the curative resection rate was 71% (22/31). The median procedure time was 25 min.

Table 3 shows the factors associated with complete and incomplete resection. The types of previous gastrectomy, tumor size, macroscopic type, and tumor histology were not associated with incomplete resection. Only the tumor's involvement with the suture lines was significantly associated with incomplete resection (*P* = 0.028).

When ESD outcomes were analyzed according to the suture line involvement (Table 4), the median procedure time was longer when the tumor involved the suture lines than when it did not (72 min versus 25 min, *P* = 0.024). Similarly, the en bloc and complete resection rates were significantly lower when the tumor involved the suture lines than when it did not (50% versus 96%, *P* = 0.037 and 25% versus 89%, *P* = 0.028, resp.). Perforation occurred during ESD in 25% (1/4) of the lesions involving the suture lines, compared with 0% (0/27) of the lesions not involving the suture lines; however, this difference did not reach statistical significance (*P* = 0.129)

### 3.3. Procedure-Related Adverse Events

The rates of procedure-related bleeding and perforation were 6% (2/31) and 3% (1/31), respectively (Table 2); no procedure-related stenosis occurred. Procedure-related bleeding was observed in 2 cases (1 adenoma and 1 cancer), and all bleeding events were successfully managed with endoscopic hemostasis. Procedure-related perforation occurred in 1 cancer case where the tumor involved the suture lines; the perforation was macroscopically detected during the procedure and the patient was successfully treated with antibiotics and restricted oral intake after clipping during the ESD procedure.

### 3.4. Follow-Up and Local Recurrence

**Figure 2:**
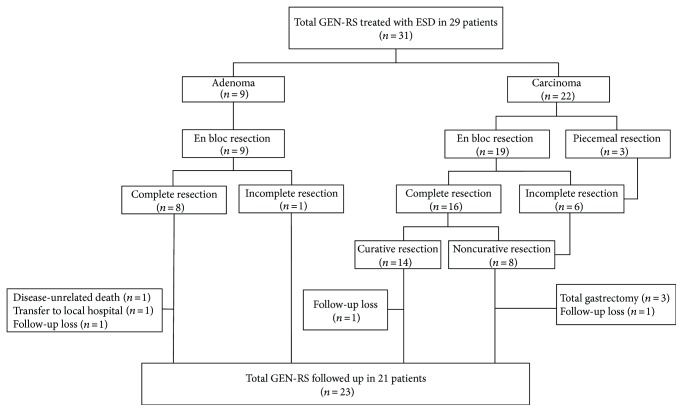
Flowchart of the patients included in the study.

Of the 9 adenomas, only 1 lesion was incompletely resected because of horizontal involvement with the tumor cells. This patient was closely observed, without additional procedures. Of the 8 patients achieving complete resection for adenomas, 1 was transferred to a local hospital and 1 was lost to follow-up. Of the 8 noncurative early cancer lesions, 2 were mucosal cancer, 1 was minute submucosal cancer, and 5 were deep submucosal cancers. Three patients with mucosal or minute submucosal cancer and positive horizontal margins were closely observed, without additional procedures. Additional surgical resection was recommended to 5 patients with deep submucosal cancer. However, 1 patient refused to undergo additional surgery and 1 was lost to follow-up. Of the 29 patients treated with ESD, 21 were followed for >6 months (Figure 2). During the median follow-up period of 25 months (range, 6–58 months), there were no cases of recurrence. One patient who achieved complete resection for an adenoma died 7 months later, due to nonprocedure-related pneumonia.

## 4. Discussion

Several clinicopathologic factors, including tumor size, tumor location, and depth of tumor invasion, contribute to the technical difficulty associated with ESD, which can influence the complete resection rate and the procedure time [18, 19]. In this respect, the remnant stomach is another difficult location for performing ESD due to the narrow inner space and the massive fibrosis along the suture lines [8]. In the present study, we demonstrated the acceptable en bloc and complete resection rates (90% and 77%, resp.) and that the therapeutic outcomes of ESD for GEN-RS were significantly influenced by the tumor's involvement with the suture lines. The present results provide endoscopists with useful information for preprocedural assessment of the difficulty of ESD for GEN-RS.

ESD for tumors in the remnant stomach, after partial gastrectomy, is technically difficult because of the limited working space for the endoscopic procedure as well as the presence of severe fibrosis and staples under the suture lines [13]. Furthermore, such lesions are generally located in the upper third of the stomach, which increases the difficulty of manipulating the endoscope and maintaining a suitable distance and direction between the lesion and the endoscope [10]. Furthermore, food residue is frequently observed in the remnant stomach, which can also hinder endoscopic procedures [20, 21]. In the present study, food residues were not identified, except in 1 patient whose procedure was postponed to the following day. Therefore, preoperative preparation involving 12 or more hours of fasting may improve the outcomes of ESD for GEN-RS.

The abovementioned anatomic features and physiologic differences of the remnant stomach can influence therapeutic ESD outcomes. In the present study, the en bloc and complete resection rates of ESD for GEN-RS were 90% and 77%, respectively, similar to the results reported in previous studies [10, 14]. However, these en bloc and complete resection rates were slightly lower compared to our previous results for ESD in the whole stomach (90% versus 97% and 77% versus 88%, resp.) [5]. Even though the number of lesions involving the suture lines was small, the en bloc and complete resection rates were lower for lesions involving the suture lines (50% and 25%, resp.). This can be explained by the fact that en bloc and complete resection rates are markedly reduced for lesions with ulcerative findings compared with lesions without such findings [22].

In a previous study of ESD for early cancer in the remnant stomach or gastric tube, a high rate of perforations (18%) was reported [13]. In that study, most of the perforations occurred in association with lesions involving the suture lines. Similarly, in the present study, perforation occurred in 1 patient whose lesion involved the suture lines. Overall, the perforation rate was only 3%, which is similar to rates reported previously (1.4%–5.6%) [10, 11]. This can be explained by the low number of lesions involving the suture lines, in the present study. However, this perforation rate is higher than that (0.4%) reported in our previous study for ESD involving the whole stomach [5]. Therefore, the endoscopist should be more careful, when performing ESD in the remnant stomach, to avoid perforations because of the previously mentioned anatomic features. In addition, delayed perforations did not occur in any case. However, the possibility of delayed perforation should not be ignored in fibrotic areas that are subjected to excessive electrocautery effects [23]. In the future, additional large studies are required to demonstrate the influence of the tumor's involvement with the suture lines on the increased risk of perforation during ESD for GEN-RS.

Despite including noncurative resection cases, there were no cases of local or extragastric recurrence during the median follow-up period of 25 months. This is consistent with previous results indicating the absence of recurrence during a median follow-up of 47.5 months [11] and a 3-year overall survival rate of 85%, with 8 deaths due to other causes and none due to gastric cancer [13]. Considering the high postoperative mortality (19%–41%) after radical total gastrectomy for cancer in the remnant stomach [24, 25] and the favorable long-term outcomes in our study, ESD appears to be an attractive alternative to completion total gastrectomy, irrespective of the type of previous gastrectomy.

The current study has several limitations. First, this study was a single-center study and is subject to the biases inherent in retrospective observational studies. We overcame these limitations to some degree because most of the ESD results were prospectively collected by the endoscopists during the procedure. Second, some technical differences existed among the three endoscopists involved in the study. These differences included their preferred knives and the time required to change the equipment. Finally, our study involved a relatively small number of patients and a short follow-up period because of the relative rarity of GEN-RS, especially after gastric conduit.

## 5. Conclusion

ESD for GEN-RS is a safe and feasible treatment, regardless of previous gastrectomy type. Considering the high morbidity and mortality associated with completion total gastrectomy and the favorable outcomes associated with endoscopic treatment, ESD may be a treatment of choice for patients with GEN-RS. However, achieving en bloc and complete resection is difficult for lesions involving the suture lines; ESD for these lesions should be carefully performed by an experienced endoscopist.

## Figures and Tables

**Table 1:** Clinicopathologic characteristics of patients with gastric epithelial neoplasms in the remnant stomach.

	Total	Distal gastrectomy	Gastric conduit
*Patient characteristics*			
Patients, *n*	29	20	9
Median age (range, years)	69 (44–80)	69 (44–80)	72 (54–78)
Sex, *n* (%)			
Male	24 (83)	17 (85)	7 (78)
Female	5 (17)	3 (15)	2 (22)
*Neoplasm characteristics*			
Lesions, *n*	31	21	10
Median tumor size (range, mm)	15 (5–40)	14 (5–30)	20 (8–40)
Macroscopic type, *n* (%)			
Elevated	19 (61)	14 (67)	5 (50)
Flat/depressed	12 (39)	7 (33)	5 (50)
Location, *n* (%)			
Involving suture lines	4 (13)	2 (10)	2 (20)
Not involving suture lines	27 (87)	19 (90)	8 (80)
Histologic type, *n* (%)			
Adenoma	9 (29)	7 (33)	2 (20)
Carcinoma	22 (71)	14 (67)	8 (80)
*Cancer characteristics*			
Lesions, *n*	22	14	8
Median tumor size (range, mm)	20 (7–40)	20 (7–30)	22.5 (8–40)
Macroscopic type, *n* (%)			
Elevated	12 (55)	7 (50)	6 (67)
Flat/depressed	10 (45)	7 (50)	3 (33)
Location, *n* (%)			
Involving suture lines	3 (14)	1 (7)	2 (25)
Not involving suture lines	19 (86)	13 (93)	6 (75)
Histologic type, *n* (%)			
Differentiated	16 (73)	10 (71)	6 (75)
Undifferentiated	6 (27)	4 (29)	2 (25)
Invasion depth, *n* (%)			
Mucosa	12 (55)	7 (50)	5 (63)
Submucosa	10 (45)	7 (50)	3 (37)

**Table 2:** Therapeutic outcomes of endoscopic submucosal dissection for gastric epithelial neoplasms in the remnant stomach.

	Total (*n* = 31)	Distal gastrectomy (*n* = 21)	Gastric conduit (*n* = 10)
En bloc resection, *n* (%)	28 (90)	19 (90)	9 (90)
Complete resection, *n* (%)	24 (77)	16 (76)	8 (80)
Causes for incomplete resection, *n* (%)	7	5	2
Piecemeal resection	3	2	1
Horizontal involvement	2	1	2
Vertical involvement	2	2	0
Curative resection^a^, *n* (%)	22 (71)	15 (71)	7 (70)
Median procedure time (min, range)	25 (4–133)	26 (4–111)	25 (13–133)
Procedure-related adverse events, *n* (%)			
Bleeding	2 (6)	1 (5)	1 (10)
Perforation	1 (3)	0 (0)	1 (10)

^a^Two completely resected cancers had deep submucosal invasion (>500 *μ*m from the muscularis mucosa).

**Table 3:** Factors for incomplete resection after endoscopic submucosal dissection for gastric epithelial neoplasms in the remnant stomach.

Factors	Complete resection (*n* = 24)	Incomplete resection (*n* = 7)	*P* value
Previous operation type, *n* (%)			1.000
Distal resection	16 (67)	5 (71)	
Gastric conduit	8 (33)	2 (29)	
Tumor size, *n* (%)			0.198
≤20 mm	15 (63)	2 (29)	
>20 mm	9 (37)	5 (71)	
Macroscopic type, *n* (%)			0.676
Elevated	16 (67)	4 (57)	
Flat/depressed	8 (33)	3 (43)	
Tumor location, *n* (%)			0.028
Involving suture line	1 (4)	3 (43)	
Not involving suture line	23 (96)	4 (57)	
Histologic type, *n* (%)			0.639
Adenoma	8 (33)	1 (14)	
Carcinoma	16 (67)	6 (86)	

**Table 4:** Therapeutic outcomes of endoscopic submucosal dissection for gastric epithelial neoplasms in the remnant stomach, according to the involvement of the suture lines.

	Involving suture lines (*n* = 4)	Not involving suture lines (*n* = 27)	*P* value
Median operation time (range, min)	72 (4–133)	25 (5–111)	0.024
En bloc resection, *n* (%)	2 (50)	26 (96)	0.037
Complete resection, *n* (%)	1 (25)	24 (89)	0.028
Procedure-related adverse events, *n* (%)
Bleeding	0 (0)	2 (7)	1.000
Perforation	1 (25)	0 (0)	0.129
